# Athletic fatigue and academic burnout in physical education students: emotional exhaustion, resilience, and latent profiles

**DOI:** 10.3389/fpsyg.2026.1855670

**Published:** 2026-06-19

**Authors:** Ziyi Zhang, Yuehan Wang, Jianing Lyu, Mingsheng Xiong

**Affiliations:** 1 School of Psychology, Wuhan Sports University, Wuhan, China; 2 Body-Brain-Mind Laboratory, School of Psychology, Wuhan Sports University, Wuhan, China

**Keywords:** academic burnout, athletic fatigue, emotional exhaustion, physical education university students, resilience

## Abstract

**Background:**

Physical education university students face the dual demands of athletic training and academic responsibilities. Athletic fatigue may be associated with academic burnout through a possible “spillover effect,” yet its underlying mechanisms, boundary conditions, and population heterogeneity remain unclear. This study integrates variable-centered and person-centered research approaches to develop and test an integrated model of the relationships between athletic fatigue and academic burnout among physical education students.

**Methods:**

A convenience sample of 539 physical education university students from a sport university in Hubei, China completed a questionnaire survey. Multi-group path analysis was used to examine the cross-group consistency of the model. Latent profile analysis was applied to identify psychological risk subgroups.

**Results:**

(1) Athletic fatigue showed a significant positive association with academic burnout. Emotional exhaustion partially mediated this relationship. Resilience significantly moderated the “emotional exhaustion → academic burnout” path, with high resilience buffering this negative effect. (2) Multi-group analysis indicated that this moderated mediation model demonstrated cross-group consistency across different sport types and athletic skill levels. (3) Latent profile analysis identified three profiles: low resilience-low exhaustion type (5.2%), general type(89.6%), and high resilience-low exhaustion type(5.2%). Notably, the low resilience-low exhaustion group exhibited the highest academic burnout despite low emotional exhaustion, a pattern that may reflect repressive coping or a dissociation between emotional awareness and actual resource depletion. Significant differences in athletic fatigue and academic burnout were found across profiles.

**Conclusion:**

Given the cross-sectional design, all findings are correlational and do not imply causation. The findings show that athletic fatigue is associated with academic burnout, that this association generalizes across groups, and they identify a high-risk “low resilience–low exhaustion” subgroup among physical education students. This provides an integrated explanation of universal patterns and individual differences, offering empirical support for targeted interventions.

## 1 Introduction

### 1.1 Research background

Physical education university students (hereafter referred to as “physical education students”) embody dual roles as both athletes and students, confronting the simultaneous demands of high-intensity training and academic tasks. Their academic burnout and mental health issues warrant particular attention (Raedeke and Smith, 2001; Schaufeli et al., 2002). Athletic fatigue (hereafter referred to as “athletic fatigue”) is a psychological syndrome resulting from long-term high-intensity training, characterized by emotional/physical exhaustion, reduced sense of accomplishment, and devaluation of sport (Raedeke and Smith, 2001; Yang and Ji, 2021). It may be transferred across domains, and this transfer is associated with academic burnout. Academic burnout refers to emotional exhaustion, cynicism toward study, and reduced academic efficacy resulting from excessive academic demands (Schaufeli et al., 2002; Lian et al., 2005). Academic burnout among physical education students has unique characteristics due to the interaction between sport and academics (Wang and Li, 2022).

It is important to clarify the conceptual boundaries among these constructs. Athletic fatigue, as measured by the Athlete Burnout Questionnaire (ABQ), is a sport-specific multidimensional syndrome encompassing emotional/physical exhaustion, reduced sense of accomplishment, and devaluation of sport. Emotional exhaustion, drawn from the Maslach Burnout Inventory-General Survey (MBI-GS), refers to general emotional resource depletion in work or study contexts. Academic burnout, adapted from Schaufeli et al., captures emotional exhaustion, cynicism, and reduced efficacy specifically toward academic tasks. Although these constructs share the dimension of “exhaustion,” they operate in distinct domains (sport vs. general vs. academic) and are measured with different instruments. This study treats them as separate but related constructs, and their discriminant validity is empirically tested in the Results section.

In recent years, researchers have explored the formation mechanisms and intervention strategies for athletic burnout from multiple perspectives, including Conservation of Resources (COR) theory (Hobfoll, 1989) and Self-Determination Theory (Madigan et al., 2021). Chinese scholars have also systematically reviewed research progress in this field (Yang and Ji, 2021). Therefore, the issue of athletic fatigue thus merits attention (Raedeke and Smith, 2001). Based on COR theory (Hobfoll, 1989), the loss and compensation of an individual's physical and psychological resources underlie stress responses and burnout. If resource loss during athletic training is unreplenished, it may be associated with poorer academic adaptation across domains. Existing studies have separately explored the causes and effects of athletic fatigue and academic burnout (Raedeke and Smith, 2001; Schaufeli et al., 2002). Still, the underlying mechanisms, boundary conditions, and cross-group stability of their relationship remain unclear, and there is a lack of person-centered heterogeneity analysis (Wang and Hanges, 2011).

Therefore, this study aims to integrate variable-centered and person-centered research approaches to construct a multilevel model, first examining emotional exhaustion as a mediating variable and resilience as a moderating variable to explore the mechanism by which athletic fatigue is associated with academic burnout among physical education students (Hu et al., 2021; Salmela-Aro and Upadyaya, 2022). Second, through a multi-group comparison, examine the cross-group consistency of this mechanism across sport types (open-skill vs. closed-skill) and athletic skill levels (National Champion, First-Grade, Second-Grade, Unranked) (Vaughan and Laborde, 2021; Chen and Wang, 2023). Finally, employ latent profile analysis to identify potential psychological risk profiles based on resilience and emotional exhaustion among physical education students (Nylund et al., 2007), and explore differences in mental health indicators across these profiles (Gustafsson et al., 2021; Zhang et al., 2024).

### 1.2 Literature review and research hypotheses

#### 1.2.1 The relationship between athletic fatigue and academic burnout

According to COR theory (Hobfoll, 1989), physical education students invest core resources such as time and energy in athletic training. Continuous resource loss triggers cross-contextual stress responses, leading to insufficient allocation of resources in the academic domain, which in turn generates academic burnout. A systematic review and meta-analysis confirmed a significant negative correlation between athletes' athletic fatigue and their academic adjustment (Madigan et al., 2021). Domestic research has also found that the level of athletic fatigue among physical education students significantly predicts their academic adjustment status, with emotion regulation playing a mediating role (Wang and Li, 2022).

From an exercise physiology perspective, athletic fatigue is not solely a psychological phenomenon but also reflects cumulative physical stress from training load, inadequate recovery, sleep disruption, and states of overreaching or non-functional overreaching. These physiological factors may interact with psychological resources (e.g., emotional exhaustion, resilience) to influence academic burnout. Although the present study focuses on psychological mechanisms, we acknowledge that physiological variables such as training load, recovery quality, and sleep play important roles in the development of athletic fatigue and should be considered in future research. In addition to resource loss, COR theory also posits resource gain spirals, where individuals can replenish lost resources through social support, positive recovery experiences, and effective coping strategies. Although the present study focuses on loss and protection mechanisms, future research should examine these gain pathways as potential buffers against the spillover of athletic fatigue to academic burnout. Based on this, this study proposes the hypothesis:

*H1: Athletic fatigue has a significant positive association with academic burnout among physical education students*.

#### 1.2.2 The mediating role of emotional exhaustion

Emotional exhaustion refers to “the overconsumption of an individual's emotional resources and feelings of athletic fatigue” (Maslach et al., 1996). Long-term high-intensity training among physical education students can easily lead to emotional resource depletion (Yang and Ji, 2021), which subsequently weakens self-regulatory abilities, making it difficult to mobilize sufficient resources to cope with academic challenges and ultimately inducing academic burnout (Salmela-Aro and Upadyaya, 2022). Longitudinal studies have shown that emotional exhaustion is a key mediating mechanism in the transfer of burnout from the athletic domain to the academic domain (Salmela-Aro and Upadyaya, 2022). Domestic scholars have also pointed out that targeted intervention strategies can help mitigate the negative impact of emotional exhaustion on academic adjustment (Li et al., 2022). Based on this, this study proposes the hypothesis:

*H2: Emotional exhaustion plays a mediating role in the association between athletic fatigue and academic burnout*.

#### 1.2.3 The moderating role of resilience

Resilience refers to “an individual's ability to cope successfully and adapt well in the face of major threats, such as adversity, trauma, and stress” (Connor and Davidson, 2003). As an important psychological resource, it can mitigate the negative effects of resource loss by mobilizing alternative resources and engaging in positive cognitive reappraisal (Xi and Zuo, 2022). Physical education students with high resilience are more likely to compensate for emotional resource depletion through emotion regulation, social support, and other means, thereby cutting off the pathway from emotional exhaustion to academic burnout. A meta-analysis further indicates that trait resilience has a stable protective effect on mental health (Hu et al., 2021). From the “resource transformation” perspective of COR theory, resilience does not directly reduce resource loss; rather, after resources have been depleted (i.e., after emotional exhaustion occurs), it blocks the pathway from depletion to negative outcomes through positive cognitive reappraisal, mobilizing social support, and meaning-making. In other words, resilience moderates “how depleted resources affect subsequent adaptation,” not “whether resources are depleted.” This theoretical positioning explains why this study places resilience on the “emotional exhaustion → academic burnout” path rather than the “athletic fatigue → emotional exhaustion” path for testing. Resilience was placed on the emotional exhaustion → academic burnout path based on the resource transformation perspective of COR theory: resilience does not prevent resource loss, but protects against the negative consequences of already depleted resources. We also tested resilience as a moderator of the athletic fatigue → emotional exhaustion path; the effect was not significant (data not shown), supporting the current model structure. Based on this, this study proposes the hypothesis:

*H3: Resilience moderates the relationship between emotional exhaustion and academic burnout*.

#### 1.2.4 The moderating role of sport type and athletic skill level

Sports can be categorized as open-skill (e.g., basketball, football) and closed-skill (e.g., track and field, swimming). Research shows that open-skill athletes need to make quick decisions in dynamically changing environments, face greater uncertainty, and use information processing methods that differ significantly from those of closed-skill athletes (Vaughan and Laborde, 2021). Domestic scholars further found that athletes in different sports differ in resilience levels, and this difference is influenced by multiple factors such as training years, competitive level, and social support (Chen and Wang, 2023). Athletic skill level reflects an athlete's competitive level. High-level athletes (National Champions, First-Grade) usually have more extensive training and competition experience and may develop stronger psychological adjustment abilities. In contrast, low-level athletes (Second-Grade, Unranked) may be more susceptible to the negative effects of athletic fatigue (Mazaheri et al., 2025). Multi-group analysis is a standard method for testing model invariance across subgroups. This study uses this method to assess the model's cross-group stability. However, whether the core pattern of associations through which athletic fatigue is associated with academic burnout varies by group or is cross-grouply stable remains empirically untested. Based on this, this study proposes the hypothesis:

*H4: The moderated mediation model demonstrates cross-group consistency across different sport types and athletic skill levels. Specifically, configural invariance (same model structure) and metric invariance (equal factor loadings) are established across groups, and no significant differences are found in path coefficients*.

#### 1.2.5 Heterogeneity of psychological risk among physical education students: a latent profile analysis perspective

Previous variable-centered research has overlooked individual heterogeneity. In contrast, person-centered latent profile analysis (LPA) can identify subgroups with similar characteristics, providing a basis for targeted interventions (Wang and Hanges, 2011). In the field of sport psychology, LPA has been used to identify heterogeneous subgroups of athletes with burnout, revealing significant differences in burnout levels among athletes with different perfectionism traits (Gustafsson et al., 2021). Domestic scholars have further applied this method to physical education student populations, discovering potential profile differences in the combination patterns of resilience and emotional exhaustion (Zhang et al., 2024), but the differences across profiles in athletic fatigue and academic burnout have not yet been verified. Based on this, this study proposes the hypothesis:

*H5: Physical education students exhibit distinct latent profiles based on resilience and emotional exhaustion, and these profiles differ significantly in academic burnout and athletic fatigue*.

## 2 Participants and methods

### 2.1 Participants

A convenience sampling method was used to select physical education university students from a university in Hubei Province as participants. A priori power analysis was conducted using GPower 3.1 software (Faul et al., 2009). Following conventions for testing moderated mediation models (Hayes, 2013) and meta-analytic results on moderation effect studies (Aguinis et al., 2005), this study adopted a conservative small effect size (*f*^2^ = 0.02) for estimation. The statistical power was set at 0.80, with a significance level α of 0.05. The calculation indicated a minimum required sample size of 395 (Cohen, 1988). Ultimately, 600 questionnaires were distributed, and 539 valid questionnaires were returned, yielding an effective response rate of 89.8%. The basic information of the research subjects is as follows: 268 males (49.72%) and 271 females (50.28%); 152 freshmen (28.2%), 136 sophomores (25.23%), 131 juniors (24.30%), and 120 seniors (22.26%); 103 master athletes (19.11%), 131 first-grade athletes (24.30%), 162 second-grade athletes (30.06%), and 143 unranked athletes (26.53%); 272 with open skills (50.46%) and 267 with closed skills (49.54%). Although the use of a single sport university limits the generalizability of findings, this sampling strategy ensured a homogeneous population of physical education students with comparable training and academic demands.

### 2.2 Measures

Athletic Fatigue Questionnaire: The Chinese version of the Athlete Burnout Questionnaire (ABQ), developed by Raedeke and Smith (2001) and validated by Liu et al. (2022), was used (Liu et al., 2022). This questionnaire consists of 15 items across three dimensions: emotional/physical exhaustion (5 items), reduced sense of accomplishment (5 items), and devaluation of sport (5 items). A 5-point Likert scale was used (1 = “Never”, 5 = “Always”). Liu et al. (2022) validated the three-factor structure of this questionnaire among Chinese collegiate athlete populations (CFI = 0.94, RMSEA = 0.06), with a total scale Cronbach's α of 0.87, dimension composite reliability (CR) ranging from 0.82 to 0.89, and average variance extracted (AVE) ranging from 0.51 to 0.63 (Liu et al., 2022). In this study, the total scale Cronbach's α coefficient was 0.89, CR was 0.91, and AVE was 0.53.

Academic Burnout Scale for University Students: The Academic Burnout Scale for University Students, developed by Lian et al. (2005), was used. This scale consists of 20 items and uses a 5-point Likert scale (1 = “Completely Disagree”, 5 = “Completely Agree”). The original scale had Cronbach's α values of 0.86, 0.79, and 0.82 for its dimensions, with a total scale Cronbach's α of 0.92, demonstrating good construct validity and criterion-related validity (Lian et al., 2005). This scale has been widely used in studies on academic adjustment among physical education student populations (Wang and Li, 2022). In this study, the total scale Cronbach's α coefficient was 0.94, CR was 0.95, and AVE was 0.58.

It is important to note that the ABQ includes an emotional/physical exhaustion subscale, which assesses sport-specific exhaustion, whereas the MBI-GS emotional exhaustion subscale measures general work/study-related emotional resource depletion. Although both tap exhaustion, they operate in different domains (sport vs. general academic context) and are validated as distinct constructs. To address potential overlap, we conducted confirmatory factor analysis comparing a model where the two exhaustion factors were combined vs. separated; the four-factor model (athletic fatigue, emotional exhaustion, resilience, academic burnout) showed superior fit, supporting their discriminant validity (see Table 1).

**Table 1:** Confirmatory factor analysis results.

Model	χ^2^	*df*	χ^2^*/df*	CFI	TLI	RMSEA	SRMR
One-factor model	1,919.692	54	35.55	0.589	0.498	0.253	0.150
Two-factor model	519.158	53	9.80	0.897	0.872	0.128	0.049
Three-factor model	119.182	51	2.34	0.985	0.981	0.050	0.036
Four-factor model	59.819	48	1.25	0.997	0.996	0.021	0.019

Emotional Exhaustion Scale: The emotional exhaustion subscale from the Maslach Burnout Inventory-General Survey (MBI-GS), developed by Maslach et al. (1996), was used, comprising 9 items (Maslach et al., 1996). This scale is specifically designed to measure feelings of emotional resource depletion in work/study contexts. A 6-point scale was used (1 = “Never”, 6 = “Every day”). Li and Shi (2003) revised the Chinese version of this scale and validated it among Chinese worker populations, reporting a Cronbach's α of 0.89 for the emotional exhaustion dimension (Li and Shi, 2003). Li et al. (2022) validated the single-factor structure of this scale among university student populations (CFI = 0.96, RMSEA = 0.07). In this study, the total scale Cronbach's α coefficient was 0.92, CR was 0.93, and AVE was 0.60.

Resilience Scale: The 10-item Connor-Davidson Resilience Scale (CD-RISC-10) developed by Connor and Davidson (2003) and revised by Campbell-Sills and Stein (2007) was used. This scale has a unidimensional structure with 10 items, using a 5-point Likert scale (1 = “Never”, 5 = “Always”). Ye et al. (2020) validated the Chinese version of this shortened scale among Chinese university student populations; confirmatory factor analysis indicated a good fit for the single-factor structure (CFI = 0.97, RMSEA = 0.05, SRMR = 0.04), with a Cronbach's α of 0.91. In this study, the total scale Cronbach's α coefficient was 0.93, CR was 0.94, and AVE was 0.62.

Demographic Variables: A self-designed questionnaire was used to collect basic information, including gender, grade, athletic skill level, training years, weekly training hours, major, and sport.

### 2.3 Procedure

Before the formal survey, a pilot study was conducted with 30 physical education students to test the readability of the questionnaires. The survey was administered online. An informed consent form was embedded on the first page, stating that clicking the “Start” button indicated that the participant had read and understood the consent information and voluntarily agreed to participate in the study. After collection, questionnaires were screened for validity. Exclusion criteria were: (a) more than 10% missing responses on any scale; (b) patterned responding (e.g., straight-lining, where the same response option was selected for more than 80% of items); (c) incorrect response to an instructed response item (e.g., “Please select ‘frequently' for this question”), which serves as an attention check. Of the 61 “excluded questionnaires, 28 had more than 10% missing responses on at least one scale, 24 showed patterned responding (straight-lining on more than 80% of items), and 9 failed the instructed response item. Thus, 539 valid questionnaires (effective response rate = 89.8%) were retained for analysis.

### 2.4 Data analysis

Step 1: Data Preprocessing. SPSS 26.0 was used for data cleaning, reverse scoring, descriptive statistics, and correlation analysis. Harman's single-factor test was employed to examine common method bias (Podsakoff et al., 2003).

Step 2: Testing Mediation and Moderation Effects. The PROCESS macro (Models 4 and 14) in SPSS 26.0 was used to test the mediating role of emotional exhaustion and the moderating role of resilience (Cohen, 1988). The bias-corrected bootstrap method with 5,000 resamples was used to calculate 95% confidence intervals for the effect sizes, which were reported using *f*^2^ and *K*^2^ (Cohen, 1988; Preacher and Kelley, 2011).

Step 3: Multi-group Path Analysis. Multi-group path analysis was conducted using Mplus 8.3, with sport type and athletic skill level as grouping variables (Wang and Hanges, 2011). A stepwise constraint approach was used to test cross-group invariance: first, configural invariance (same model structure across groups), second, measurement invariance (equal factor loadings across groups), and finally, path coefficient invariance (equal path coefficients across groups). Model comparison criteria were: ΔCFI ≤ 0.01, ΔRMSEA ≤ 0.015, ΔSRMR ≤ 0.030 for invariance to hold (Chen, 2007).

Step 4: Latent Profile Analysis. Latent profile analysis was performed in Mplus 8.3 using standardized scores of resilience and emotional exhaustion as indicators, estimating models with 1 to 5 classes sequentially (Nylund et al., 2007). Models were estimated using robust maximum likelihood (MLR). Model fit evaluation comprehensively considered: Akaike Information Criterion (AIC), Bayesian Information Criterion (BIC), and sample-size-adjusted BIC (aBIC), where smaller values indicate better fit; Entropy > 0.80 indicates good classification accuracy; significant *p*-values (*p* < 0.05) for Lo-Mendell-Rubin (LMR) test and Bootstrap Likelihood Ratio Test (BLRT) indicate that a k-class model is better than a k-1 class model. Practical utility and theoretical interpretability were also considered, and classes with sample proportions below 5% were excluded. After determining the optimal model, the BCH method was used to test differences in outcome variables across profiles, and the DCAT method was used to test differences in the distributions of demographic variables across profiles (Zhang et al., 2024). Because all constructs were measured with single observed variables (scale means), there are no factor loadings to constrain across groups; therefore, traditional measurement invariance (i.e., equal factor loadings) is not applicable. We therefore tested only configural invariance (same model structure) and path coefficient invariance (equal regression coefficients). The cutoff criteria for model comparison (ΔCFI ≤ 0.01, ΔRMSEA ≤ 0.015, ΔSRMR ≤ 0.030) were adopted from Chen (2007) for configural and path coefficient invariance testing, acknowledging that these criteria are originally developed for latent variable models but used here as a heuristic.

## 3 Results and analysis

### 3.1 Common method bias test and confirmatory factor analysis

Harman's single-factor test was conducted by performing unrotated principal component analysis on all 54 items. The results showed that the first factor explained 30.80% of the variance, which is below the critical threshold of 40% (Podsakoff et al., 2003), indicating that common method bias was within an acceptable range.

Confirmatory factor analysis was performed using Mplus to test the discriminant validity of the four core variables. The item parceling method was used to aggregate items within each dimension into 12 observed indicators. Four nested models were compared, and the results are shown in Table 1. The four-factor model demonstrated the best fit indices (χ^2^ (48) = 59.819, CFI = 0.997, TLI = 0.996, RMSEA = 0.021, SRMR = 0.019) and was significantly superior to other models, indicating good discriminant validity among the four variables.

To examine whether item parceling inflated model fit, we also conducted CFA using all 54 individual items (without parceling). The model fit was lower but still marginal (CFI = 0.821, RMSEA = 0.063, SRMR = 0.056), suggesting that parceling may have improved fit indices. However, the four-factor structure remained largely consistent, and discriminant validity (Fornell-Larcker) continued to hold with item-level data (available upon request).

Discriminant validity was assessed using the Fornell-Larcker criterion. The square root of the average variance extracted (AVE) for each construct exceeded its correlations with all other constructs: athletic fatigue (√AVE = 0.73, maximum |*r*| = 0.69), emotional exhaustion (√AVE = 0.78, maximum |*r*| = 0.67), resilience (√AVE = 0.79, maximum |*r*| = 0.22), and academic burnout (√AVE = 0.76, maximum |*r*| = 0.67). These results support the discriminant validity of the four constructs.

To assess multicollinearity, we calculated variance inflation factors (VIFs) for all predictor variables in the regression models. All VIFs were below 2 (range = 1.05–1.93), indicating no serious multicollinearity.

### 3.2 Descriptive statistics and correlation analysis of variables

Pearson correlation analysis was used to examine the relationships among variables. Athletic fatigue was significantly positively correlated with academic burnout (*r* = 0.649, *p* < 0.01) and emotional exhaustion (*r* = 0.685, *p* < 0.01), and significantly negatively correlated with resilience (*r* = −0.159, *p* < 0.01). Academic burnout was significantly positively correlated with emotional exhaustion (*r* = 0.672, *p* < 0.01) and significantly negatively correlated with resilience (*r* = −0.199, *p* < 0.01). Emotional exhaustion was significantly negatively correlated with resilience (*r* = −0.218, *p* < 0.01). These results provided a foundation for testing the mediation and moderation effects.

The relatively high correlations among athletic fatigue, emotional exhaustion, and academic burnout (ranging from 0.65 to 0.69) raise the possibility of construct overlap. This is expected given that these constructs share a common core of exhaustion experience, although they operate in different domains (sport vs. general vs. academic).

### 3.3 Testing the mediating effect of emotional exhaustion

Using the SPSS PROCESS macro (Model 4) developed by Hayes (2013), the mediating effect of emotional exhaustion between athletic fatigue and academic burnout was tested, controlling for gender, grade, and training hours. The results are shown in Table 2. The total association of athletic fatigue with academic burnout was significant (β = 0.65, *t*_(535)_ = 19.76, *p* < 0.001, 95% CI = [0.66, 0.80]), supporting H1. Given the cross-sectional design, this reflects a correlational relationship. Athletic fatigue significantly positively predicted emotional exhaustion (β = 0.69, *t*_(535)_ = 21.81, *p* < 0.001, 95% CI = [0.94, 1.13]); emotional exhaustion significantly positively predicted academic burnout (β = 0.43, *t*_(535)_ = 10.43, *p* < 0.001, 95% CI = [0.26, 0.38]); after controlling for emotional exhaustion, the direct effect of athletic fatigue on academic burnout remained significant but weakened (β = 0.35, *t*_(535)_ = 8.62, *p* < 0.001, 95% CI = [0.31, 0.49]).

**Table 2:** Mediating effect analysis of emotional exhaustion.

Path	Effect type	β	*B*	*SE*	*t*	95%*CI*	Proportion
Athletic fatigue → Academic burnout	Total effect (c)	0.65	0.73	0.04	19.76^***^	[0.66, 0.80]	100%
Athletic fatigue → Emotional exhaustion	a path	0.69	1.04	0.05	21.81^***^	[0.94, 1.13]	-
Emotional exhaustion → academic burnout	b path	0.43	0.32	0.03	10.43^***^	[0.26, 0.38]	-
Athletic fatigue → academic burnout	Direct effect (c')	0.35	0.40	0.05	8.62^***^	[0.31, 0.49]	54.8%
Athletic fatigue → academic burnout	Indirect effect (a × b)	—	0.33	0.04	—	[0.26, 0.40]	45.2%

^***^p < 0.001.

β, standardized coefficient; *B*, unstandardized coefficient; SE, standard error; Bootstrap resampling, 5,000 times; CI, confidence interval.

The bootstrap method was used to test the mediating effect. The indirect effect of emotional exhaustion was significant (indirect effect = 0.33, Boot SE = 0.04, 95% CI = [0.26, 0.40]), accounting for 45.2% of the total effect. The completely standardized indirect effect *K*^2^ = 0.293, which, according to Preacher and Kelley's (2011) criteria, is considered a large effect. Therefore, emotional exhaustion partially mediated the relationship between athletic fatigue and academic burnout, supporting H2.

### 3.4 Testing the moderating effect of resilience

#### 3.4.1 Moderation effect

The SPSS PROCESS macro (Model 14) was used to test the moderating effect of resilience on the “emotional exhaustion → academic burnout” path. Emotional exhaustion and resilience were standardized before analysis, and the interaction term (emotional exhaustion × resilience) was computed. The results are shown in Table 3.

**Table 3:** Testing the moderated mediation model.

Variable	*B*	*SE*	*t*	*p*	95% CI
Athletic fatigue	0.424	0.038	11.24	<0.001	[0.350, 0.498]
Emotional exhaustion	0.310	0.025	12.33	<0.001	[0.261, 0.360]
Resilience	0.973	0.064	15.15	<0.001	[0.847, 1.099]
Emotional exhaustion × resilience	−0.295	0.018	−16.38	<0.001	[−0.330, −0.260]

^***^p < 0.001.

B, unstandardized coefficient; SE, standard error; CI, confidence interval.

The moderated mediation model fit well, explaining 68.12% of the variance in academic burnout (*R*^2^ = 0.681, *F*_(4, 534)_ = 285.25, *p* < 0.001). The direct effect of athletic fatigue on academic burnout was significant (B = 0.424, *t*_(534)_ = 11.24, *p* < 0.001). The main effect of emotional exhaustion on academic burnout was significant (B = 0.310, *t*_(534)_ = 12.33, *p* < 0.001). The interaction term between emotional exhaustion and resilience significantly predicted academic burnout (B = −0.295, *t*_(534)_ = −16.38, *p* < 0.001, 95% CI = [−0.330, −0.260]). After adding the interaction term, the model's explanatory power significantly increased (Δ*R*^2^ = 0.160, *p* < 0.001), with a moderation effect size *f*^2^ = 0.334 (calculated as *f*^2^ = Δ*R*^2^/(1-*R*^2^_base), where *R*^2^_base = 0.521), which is considered a medium-to-large effect (Cohen, 1988). Therefore, resilience significantly moderated the relationship between emotional exhaustion and academic burnout, supporting H3.

Note that the positive main effect of resilience (B = 0.973, *p* < 0.001) in Table 3 should not be interpreted as a direct effect. In a model with an interaction term (emotional exhaustion × resilience), the main effect of resilience represents its effect when emotional exhaustion is zero (i.e., at the mean after centering). Given that resilience was centered at its mean, this positive coefficient simply reflects that, at average levels of emotional exhaustion, higher resilience was unexpectedly associated with higher academic burnout. However, the significant negative interaction (B = −0.295) qualifies this main effect, indicating that the relationship between resilience and academic burnout depends on the level of emotional exhaustion. Simple slopes analysis (Section 3.4.2) shows that high resilience buffers the negative impact of emotional exhaustion, which is the theoretically meaningful finding. In other words, the protective role of resilience is reflected in the significant negative interaction term, not in the main effect. The positive main effect merely represents the simple effect when emotional exhaustion is at its centered mean (i.e., zero).

**Figure 1:**
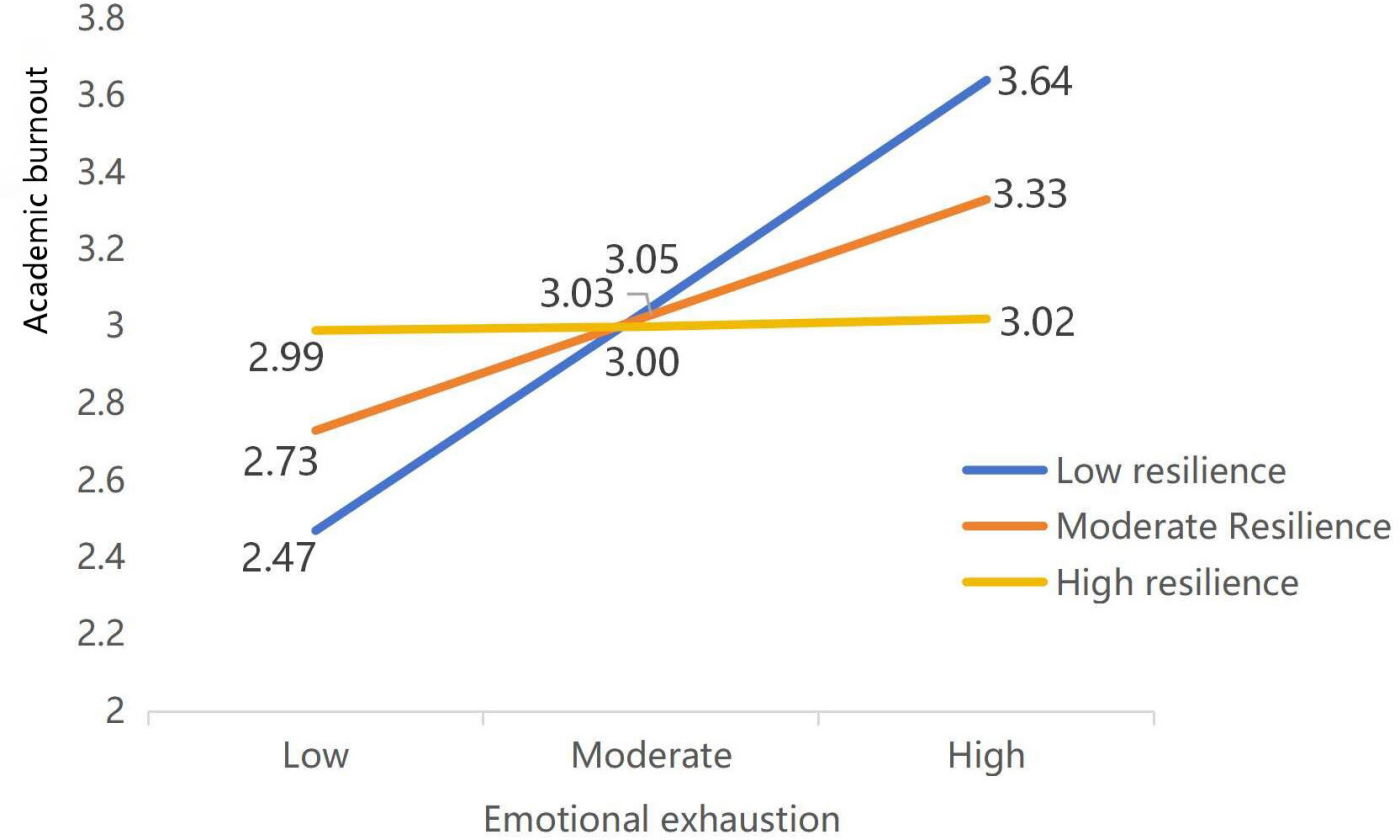
Simple slopes analysis of emotional exhaustion on academic burnout at different levels of resilience.

A graphical representation of the simple slopes (Figure 1) shows that the protective effect of resilience only emerges at higher levels of emotional exhaustion, which explains why the main effect of resilience (at mean exhaustion) appears positive.

#### 3.4.2 Simple slopes analysis

To reveal the nature of the interaction, resilience was divided into high, medium, and low groups based on one standard deviation above and below the mean (M ± 1 SD), and the predictive effect of emotional exhaustion on academic burnout was examined across these groups. The results are shown in Table 4 and Figure 1.

**Table 4:** Simple slopes analysis at different levels of resilience.

Resilience level	Effect value	*SE*	*t*	*p*	95% CI
Low resilience (*M*- 1SD)	0.605	0.031	19.55	<0.001	[0.544, 0.665]
Moderate Resilience (*M* = 0)	0.310	0.025	12.33	<0.001	[0.261, 0.360]
High resilience (*M* + 1SD)	0.016	0.031	0.50	0.616	[−0.045, 0.076]

^***^p < 0.001.

M, mean; SD, standard deviation; SE, standard error; CI, confidence interval.

For physical education students with low resilience (M - 1SD), the positive predictive effect of emotional exhaustion on academic burnout was strong (simple slope = 0.605, *t*_(534)_ = 19.55, *p* < 0.001), with an effect size *f*^2^ = 0.72 (large effect). For students with high resilience (M + 1SD), the positive predictive effect of emotional exhaustion on academic burnout was not significant (simple slope = 0.016, *t*_(534)_ = 0.50, *p* = 0.616), with an effect size *f*^2^ ≈ 0.0005 (negligible effect). The between-group difference effect size was *f*^2^ = 1.20 (large effect). This indicates that as resilience increases, the positive association between emotional exhaustion and academic burnout gradually weakens. When resilience is low, emotional exhaustion is associated with higher academic burnout; when resilience is high, this effect completely disappears. Therefore, resilience buffers the negative impact of emotional exhaustion on academic burnout, supporting H3.

#### 3.4.3 Testing the moderated mediation effect

Further analysis examined whether the mediating effect of emotional exhaustion between athletic fatigue and academic burnout differed across levels of resilience. Using PROCESS Model 14, conditional indirect effects were calculated at three levels of resilience (M - 1 SD, M, M + 1 SD). The results are shown in Table 5.

**Table 5:** Conditional indirect effects at different levels of resilience.

Resilience level	Indirect effect	Boot SE	Boot LLCI	Boot ULCI
Low resilience (*M*- 1*SD*)	0.627	0.039	0.552	0.704
Moderate resilience (*M* = 0)	0.322	0.028	0.268	0.377
High resilience (*M* + 1SD)	0.016	0.032	−0.046	0.080
Index of moderated mediation	−0.306	0.022	−0.348	−0.263

Boot SE, bootstrap standard error; Boot LLCI and Boot ULCI, Bootstrap lower and upper limits of the 95% confidence interval; Bootstrap resampling, 5,000 times.

As the resilience level increased, the mediating effect of emotional exhaustion gradually weakened. The indirect effect was significant in the low resilience group (indirect effect = 0.627, 95% CI = [0.552, 0.704], effect size *K*^2^ = 0.52, large effect) and the medium resilience group (indirect effect = 0.322, 95% CI = [0.268, 0.377], effect size *K*^2^ = 0.27, medium effect); the indirect effect was not significant in the high resilience group (indirect effect = 0.016, 95% CI = [−0.046, 0.080], effect size *K*^2^ ≈ 0.01, negligible effect). The index of moderated mediation was −0.306 (Boot SE = 0.022, 95% CI = [−0.348, −0.263], effect size *f*^2^ = 0.09, medium effect). This confirms that resilience significantly moderates the mediating effect of emotional exhaustion and that high resilience can eliminate it.

### 3.5 Multi-group path analysis

#### 3.5.1 Using sport type as the grouping variable

The multi-group path analysis model using sport type as the grouping variable showed a good fit (χ^2^(8) = 9.12, CFI = 0.998, TLI = 0.998, RMSEA = 0.023, SRMR = 0.062), supporting configural invariance. Because all constructs were measured with single observed variables (scale means), there are no factor loadings to constrain across groups; therefore, traditional measurement invariance (i.e., equal factor loadings) is not applicable. We therefore tested only configural invariance (same model structure) and path coefficient invariance (equal regression coefficients). The results are shown in Table 6. The fully constrained model (forcing path coefficients to be equal across the two groups) did not differ significantly from the freely estimated model (Δχ^2^(4) = 5.50, *p* > 0.05), and changes in CFI, RMSEA, and SRMR were all below recommended thresholds (ΔCFI ≤ 0.01, ΔRMSEA ≤ 0.015, ΔSRMR ≤ 0.030), supporting path coefficient invariance. While these thresholds are commonly used for latent variable models, we adopt them here as a heuristic guide for observed variable path analysis. The absolute values of Cohen's *d* effect sizes for between-group differences in path coefficients ranged from 0.02 to 0.11, all below the small-effect threshold of 0.20, indicating cross-group consistency of the model across different sport-type groups.

**Table 6:** Comparison of path coefficients across different sport type groups.

Path	Open skill (*n* = 272)			Closed skill (*n* = 267)		
	β	*SE*	*p*	β	*SE*	*p*
a (Athletic fatigue → emotional exhaustion)	0.662	0.024	<0.001	0.676	0.024	<0.001
b (Emotional exhaustion → academic burnout)	0.414	0.033	<0.001	0.410	0.032	<0.001
c' (Direct effect)	0.372	0.033	<0.001	0.376	0.033	<0.001
γ (Emotional exhaustion × resilience)	−0.400	0.029	<0.001	−0.392	0.029	<0.001

^***^p < 0.001.

β, standardized coefficient; SE, standard error.

#### 3.5.2 Using athletic skill level as the grouping variable

The multi-group path analysis model using athletic skill level as the grouping variable showed a good fit (χ^2^(32) = 40.64, CFI = 0.993, TLI = 0.992, RMSEA = 0.045, SRMR = 0.076), supporting configural invariance. Because all constructs were measured with single observed variables (scale means), there are no factor loadings to constrain across groups; therefore, traditional measurement invariance (i.e., equal factor loadings) is not applicable. We therefore tested only configural invariance (same model structure) and path coefficient invariance (equal regression coefficients). The results are shown in Table 7. The fully constrained model (forcing path coefficients to be equal across the four groups) did not differ significantly from the freely estimated model (Δχ^2^(12) = 15.73, *p* = 0.203), and changes in CFI, RMSEA, and SRMR were all below recommended thresholds (ΔCFI ≤ 0.01, ΔRMSEA ≤ 0.015, ΔSRMR ≤ 0.030), supporting path coefficient invariance. While these thresholds are commonly used for latent variable models, we adopt them here as a heuristic guide for observed variable path analysis. Neither sport type nor athletic skill level significantly moderated the moderated mediation model, supporting H4.

**Table 7:** Comparison of path coefficients across different athletic skill level groups.

Path	National champion (*n* = 103)	First-grade (*n* = 131)	Second-grade (*n* = 162)	Unranked (*n* = 143)
	β (*SE*)	β (*SE*)	β (*SE*)	β (*SE*)
a (Athletic fatigue → emotional exhaustion)	0.628 (0.038)^***^	0.673 (0.034)^***^	0.693 (0.031)^***^	0.670 (0.033)^***^
b (Emotional exhaustion → academic burnout)	0.403 (0.036)^***^	0.390 (0.033)^***^	0.411 (0.033)^***^	0.413 (0.033)^***^
c' (Direct effect)	0.355 (0.033)^***^	0.368 (0.034)^***^	0.400 (0.035)^***^	0.388 (0.034)^***^
γ (Emotional exhaustion × resilience)	−0.456 (0.041)^***^	−0.417 (0.042)^***^	−0.383 (0.043)^***^	−0.366 (0.042)^***^

^***^p < 0.001.

β, standardized coefficient; SE, standard error.

#### 3.5.3 Conditional indirect effect analysis

Conditional indirect effects at different resilience levels were examined across groups, with results shown in Table 8. The moderation pattern was highly consistent across groups: the indirect effect was significant in the low resilience group (0.600–0.603); remained significant but weakened in the medium resilience group (0.308–0.314); and was not significant in the high resilience group (0.015–0.025). This result further validates that the “buffer” effect of resilience has cross-group stability.

**Table 8:** Conditional indirect effects at different levels of resilience.

Resilience level	Open skill		National champion	
	Indirect effect	95% CI	Indirect effect	95%CI
Low resilience (*M*- 1*SD*)	0.603^***^	[0.529, 0.681]	0.600^***^	[0.529, 0.678]
Moderate resilience (*M* = 0)	0.314^***^	[0.261, 0.371]	0.308^***^	[0.255, 0.364]
High resilience (*M* + 1*SD*)	0.025	[−0.037, 0.087]	0.015	[−0.042, 0.075]

^***^p < 0.001.

CI, confidence interval.

### 3.6 Latent profile analysis

#### 3.6.1 Model fit and determination of number of classes

Using standardized scores of resilience and emotional exhaustion as indicators, models with 1 to 5 classes were estimated sequentially. The selection of resilience and emotional exhaustion as LPA indicators was theory-driven, as these two variables represent core psychological resources and depletion status, which jointly define risk profiles according to COR theory. Model fit indices are shown in Table 9. AIC, BIC, and aBIC decreased as the number of classes increased. LMR and BLRT were significant for models with 2 to 4 classes (*p* < 0.001), but the smallest class proportion dropped to 3.0% for the 4-class model. The 5-class model showed a warning for the BLRT test, and the smallest class proportion dropped to 2.8%, below the acceptable threshold of 3%. The 3-class model had an entropy of 0.907 (>0.8), the smallest class proportion of 5.2% (>5%), was parsimonious, and the classes had clear theoretical meaning. Considering all factors, the 3-class model was determined as the optimal solution.

**Table 9:** Comparison of model fit indices for latent profile analysis.

Model	Parameters	LogL	AIC	BIC	aBIC	Entropy	LMR *P*	BLRT *p*	Smallest class proportion
1 class	4	−1,533.518	3,075.036	3,092.195	3,079.497	—	—	—	100.0%
2 class	7	−1,504.799	3,023.597	3,053.625	3,031.405	0.914	0.000	0.000	4.8%
3 class	10	−1,474.619	2,969.237	3,012.134	2,980.391	0.907	0.000	0.000	5.2%
4 class	13	−1,450.060	2,926.119	2,981.886	2,940.619	0.908	0.000	0.000	3.0%
5 class	16	−1,424.148	2,880.297	2,948.932	2,898.143	0.910	0.000	0.000^a^	2.8%

LogL, log-likelihood; AIC, akaike information criterion; BIC, Bayesian information criterion; aBIC, sample-size-adjusted BIC; LMR, Lo–Mendell–Rubin test; BLRT, Bootstrap likelihood ratio test.

^a^The BLRT test for the 5-class model yielded a warning: “*P*-value may not be trustworthy due to local maxima,” suggesting potentially unreliable results.

#### 3.6.2 Characteristics and naming of the three latent profiles

**Figure 2:**
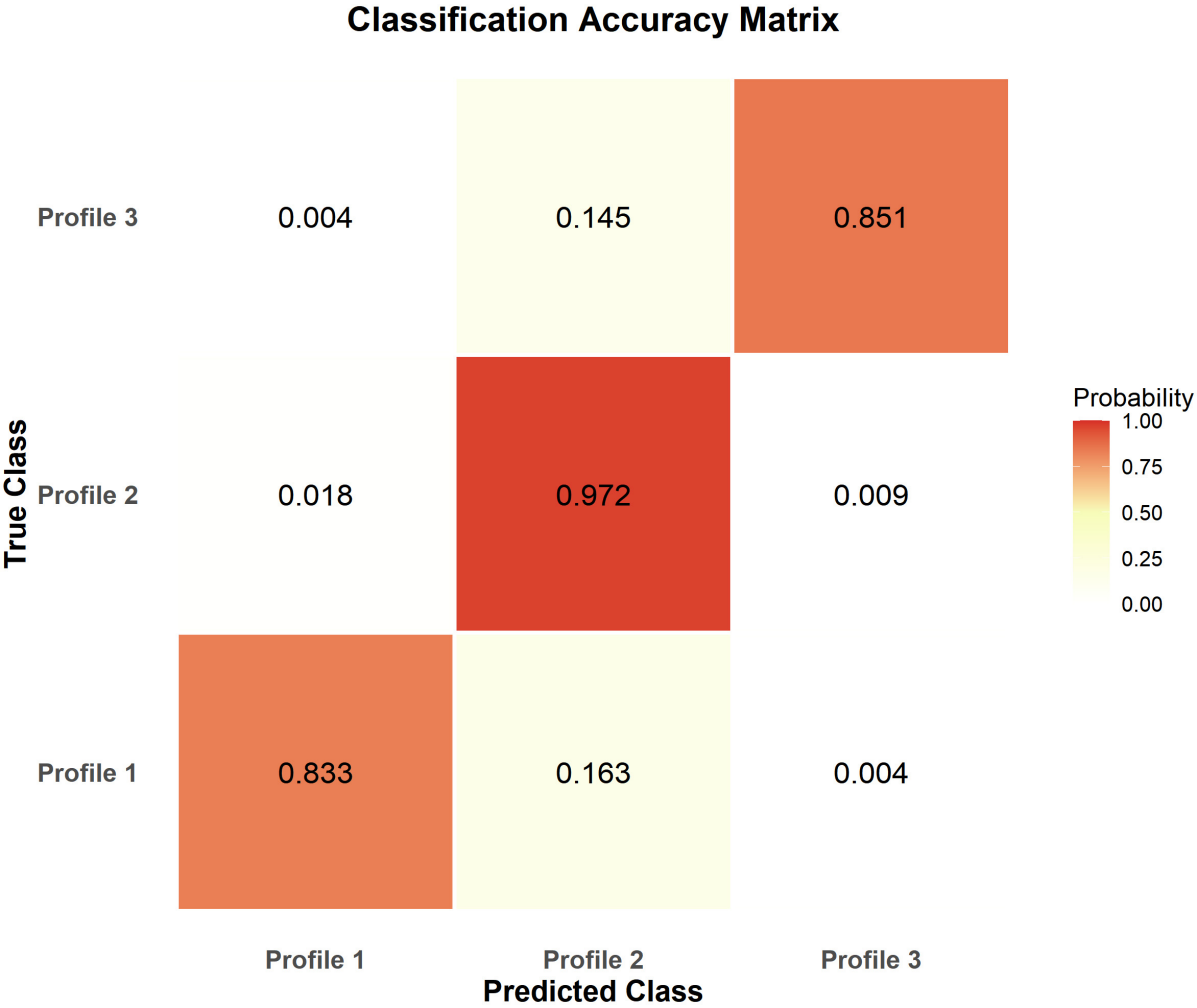
Classification accuracy matrix.

To assess the classification accuracy of the 3-class model, the average latent class membership probabilities were computed, with the results shown in Figure 2. Diagonal values represent the average probability that individuals are correctly classified into their respective profiles, while off-diagonal values represent misclassification probabilities.

##### 3.6.2.1 Additional diagnostics and sensitivity analyses

The average posterior probabilities for correct classification were 0.833, 0.972, and 0.851 for Profiles 1, 2, and 3, respectively, all exceeding the recommended threshold of 0.80, indicating good classification accuracy (Nylund et al., 2007). To assess the stability of the 3-class solution, we conducted sensitivity analyses using increased random starts (STARTS = 1000 250 with 20 initial stage iterations). The best loglikelihood value (−1,474.619) was replicated 8 times across the 1,000 random starts, and the model terminated normally. The class proportions and profile characteristics (e.g., Z-scores for resilience and emotional exhaustion) remained nearly identical to those obtained with the original random start specification. The unusual pattern of the low resilience-low exhaustion group (low exhaustion but high burnout) was consistently identified, supporting the robustness of this finding.

To examine whether training load (weekly training hours) explains the low resilience-low exhaustion pattern, we conducted an LPA with training hours as a covariate using the R3STEP method. Results showed that training hours did not significantly predict membership in the low resilience-low exhaustion group compared to the high resilience-low exhaustion group (B = 0.220, SE = 0.306, *p* = 0.473). Thus, training load alone does not account for the atypical subgroup.

#### 3.6.3 Characteristics and naming of the three latent profiles

**Figure 3:**
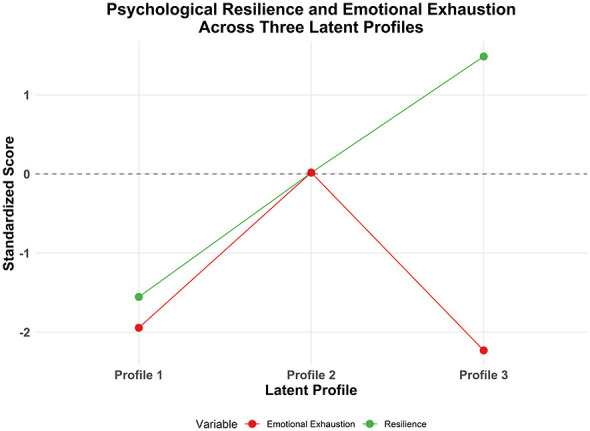
Characteristics of resilience and emotional exhaustion for the three latent profiles.

Based on the 3-class model's estimation results, the conditional means and characteristics of the three latent profiles are shown in Table 10 and Figure 3. The characteristics of the three profiles are as follows:

**Table 10:** Distribution of demographic variables across profiles.

Variable	Profile 1	Profile 2	Profile 3	χ^2^	*p*
Sport skill type				0.301	0.860
Open skill	48.8%	53.4%	51.3%		
Closed skill	51.2%	46.6%	48.7%		
Athletic level				0.699	0.705
National champion/first-grade	58.7%	49.7%	53.4%		
Second-grade/unranked	41.3%	50.3%	46.6%		
Grade				3.901	0.690
Freshman	14.3%	20.6%	17.9%		
Sophomore	30.9%	24.3%	27.6%		
Junior	32.1%	28.7%	16.3%		
Senior	22.7%	26.5%	38.2%		

Profile 1 (Low Resilience-Low Exhaustion Type, *n* = 28, 5.2%): Extremely low resilience level (Zres = −1.554, *p* < 0.001), and extremely low emotional exhaustion (Zee = −1.944, *p* < 0.001). This group lacks psychological resources, but emotional resources have not yet been severely depleted, potentially being in an early stage of psychological risk.

Profile 2 (General Type, *n* = 483, 89.6%): Both resilience (Zres = 0.016, *p* > 0.05) and emotional exhaustion (Zee = 0.019, *p* > 0.05) are close to the overall average. This group represents the main population with a relatively stable psychological status.

Profile 3 (High Resilience-Low Exhaustion Type, *n* = 28, 5.2%): Extremely high resilience level (Zres = 1.486, *p* < 0.001), and extremely low emotional exhaustion (Zee = −2.231, *p* < 0.001). This group has the best psychological adaptation.

#### 3.6.4 Testing differences in outcome variables across profiles

The BCH method was used to test differences in academic burnout and athletic fatigue across the three profiles. The results are shown in Table 11.

**Table 11:** BCH difference tests for outcome variables across profiles.

Variable	Profile	M	SE	Overall test	Pairwise comparison	χ^2^	*p*	*Cohen's d*
Academic burnout				χ^2^ = 399.537^***^				
	Profile 1	5.014	0.137		Profile 1 vs. Profile 2	127.814	<0.001	0.85
	Profile 2	3.379	0.042		Profile 1 vs. Profile 3	380.736	<0.001	1.10
	Profile 3	1.542	0.113		Profile 2 vs. Profile 3	225.859	<0.001	0.78
Athletic fatigue				χ^2^ = 175.485^***^				
	Profile 1	4.509	0.110		Profile 1 vs. Profile 2	169.395	<0.001	0.62
	Profile 2	3.007	0.033		Profile 1 vs. Profile 3	108.656	<0.001	0.48
	Profile 3	2.872	0.112		Profile 2 vs. Profile 3	1.289	0.256	0.05

M, mean; SE, standard error. Profile 1 = low resilience–low exhaustion type; Profile 2 = general type; Profile 3 = high resilience–low exhaustion type; Cohen's *d* is the effect size; according to Cohen's (1988) criteria, 0.02 = small effect, 0.50 = medium effect, 0.80 = large effect. ^***^p < 0.001.

Academic Burnout: Significant differences were found among the three groups (χ^2^(2) = 399.537, *p* < 0.001), with an effect size η^2^ = 0.43 (large effect). *Post-hoc* comparisons revealed all pairwise comparisons were significant: Profile 1 had the highest academic burnout level (M = 5.014), significantly higher than Profile 2 (M = 3.379, χ^2^(1) = 127.814, *p* < 0.001, Cohen's *d* = 0.85) and Profile 3 (M = 1.542, χ^2^(1) = 380.736, *p* < 0.001, Cohen's *d* = 1.10); Profile 2 was significantly higher than Profile 3 (χ^2^(1) = 225.859, *p* < 0.001, Cohen's *d* = 0.78). This result indicates that low resilience, even when not accompanied by high emotional exhaustion, is already associated with the highest academic burnout, suggesting an independent protective role of resilience in academic adaptation.

Athletic Fatigue: Significant differences were found among the three groups (χ^2^(2) = 175.485, *p* < 0.001), with an effect size η^2^ = 0.25 (large effect). *Post-hoc* comparisons revealed that Profile 1 had the highest athletic fatigue level (M = 4.509), significantly higher than Profile 2 (M = 3.007, χ^2^(1) = 169.395, *p* < 0.001, Cohen's *d* = 0.62) and Profile 3 (M = 2.872, χ^2^(1) = 108.656, *p* < 0.001, Cohen's *d* = 0.48); however, there was no significant difference between Profile 2 and Profile 3 (χ^2^(1) = 1.289, *p* = 0.256, Cohen's *d* = 0.05).

This result suggests that low resilience is an important risk factor for athletic fatigue. In contrast, the protective effect of high resilience against athletic fatigue may be weaker than its protective effect against academic burnout. Therefore, significant differences exist across profiles in academic burnout and athletic fatigue, supporting H5.

#### 3.6.5 Differences in demographic variable distribution across profiles

The DCAT method was used to test differences in the distribution of demographic variables across the three profiles. The results are shown in Table 10. There were no significant differences among the three profiles in sport type (χ^2^(2) = 0.301, *p* = 0.860, η^2^ = 0.0006), athletic skill level (χ^2^(2) = 0.699, *p* = 0.705, η^2^ = 0.001), or grade (χ^2^(6) = 3.901, *p* = 0.690, η^2^ = 0.007). The different combinations of resilience and emotional exhaustion demonstrate cross-group stability; mental health interventions should target all physical education students, and the basis for targeted screening and precision interventions should be the individual's psychological characteristics.

### 3.7 Summary of hypothesis testing results

H1: Athletic fatigue shows a significant positive association with academic burnout—Supported.
H2: Emotional exhaustion mediates the relationship between athletic fatigue and academic burnout—Supported (partial mediation).
H3: Resilience moderates the relationship between emotional exhaustion and academic burnout—Supported.
H4: The moderated mediation model demonstrates cross-group consistency across different sport types and athletic skill levels—Supported.
H5: Different psychological risk profiles exist, and these profiles differ in outcome variables—Supported.

## 4 Discussion

### 4.1 The core mechanism of athletic fatigue's impact on academic burnout

This study found a positive association between athletic fatigue and academic burnout among physical education students (H1), which is consistent with the cross-contextual spillover effect of resource loss in COR theory—the continuous loss of physical and psychological resources during athletic training is associated with insufficient resource investment in the academic domain, which may subsequently relate to academic burnout.

Emotional exhaustion partially mediated the relationship between athletic fatigue and academic burnout (H2), with the indirect effect accounting for 45.2% of the variance (*K*^2^ = 0.293). This suggests that athletic fatigue is directly associated with academic burnout, and also indirectly via emotional exhaustion. Emotional resource depletion caused by long-term high-intensity training weakens physical education students' self-regulatory abilities, making it difficult for them to cope with academic stress. This result aligns with the longitudinal study findings of Salmela-Aro and Upadyaya (2022), further suggesting that emotional exhaustion may play a key mediating role in the transfer of burnout from the athletic domain to the academic domain.

Resilience significantly moderated the “emotional exhaustion → academic burnout” path (H3), with an interaction term β = −0.295 (*p* < 0.001) and a moderation effect size *f*^2^ = 0.334. Simple slopes analysis revealed that at low resilience levels, the predictive effect of emotional exhaustion on academic burnout was strong (simple slope = 0.605); at high resilience levels, this predictive effect completely disappeared (simple slope = 0.016). This result supports the core tenet of COR theory that “psychological resources can compensate for resource loss”: physical education students with high resilience can compensate for emotional resource depletion through positive cognitive reappraisal, mobilizing social support, meaning-making, etc., thereby blocking the transformation from emotional exhaustion to academic burnout. In contrast, those with low resilience lack effective resource compensation strategies, amplifying the negative impact of emotional exhaustion.

### 4.2 Robustness of the mechanism: generalisability across sport type and athletic skill level

This study found that the moderated mediation model demonstrated high cross-group consistency across different sport types and athletic skill levels (H4). That is, the core pattern of associations between athletic fatigue and academic burnout did not differ across these subgroups. This result enhances the external validity of COR theory, showing that its core logic of resource loss and compensation generalizes across different subgroups of physical education students. Although open-skill and closed-skill athletes differ in information processing styles, and athletes of different skill levels vary in psychological adjustment abilities (Chen and Wang, 2023; Nylund et al., 2007), the associations among athletic fatigue, emotional exhaustion, resilience, and academic burnout remained stable. This provides a basis for developing standardized mental health intervention programs for this population.

### 4.3 Heterogeneity of the population: low resilience-low exhaustion as a high-risk “invisible group”

This study found significant heterogeneity among physical education students in resilience and emotional exhaustion, identifying three latent profiles (H5). This finding complements previous variable-centered research: variable-centered studies revealed an overall negative correlation between resilience and emotional exhaustion (Halbesleben et al., 2024), while the person-centered study found that this relationship manifests as different combination patterns across subgroups.

This low resilience-low exhaustion pattern does not fully align with the direct resource loss pathway in COR theory, but several plausible explanations exist. First, it may reflect repressive coping or response bias: individuals with low resilience may underreport negative emotions while still experiencing internal resource depletion. Second, unmeasured factors such as perceived stress, coping styles, or academic pressure may directly contribute to burnout independent of reported exhaustion. Third, cultural norms of endurance and restraint (Li and Wang, 2023) may reduce emotional expression but not eliminate burnout symptoms.

Why would low self-reported exhaustion co-occur with high burnout? A key mechanism may be dissociation between emotional awareness and actual resource depletion. Individuals with low resilience often adopt repressive coping, actively avoiding or denying negative internal states (e.g., fatigue, frustration). This strategy temporarily masks the subjective experience of exhaustion, allowing them to continue training and studying without conscious awareness of resource drain. However, the underlying depletion persists, eventually manifesting as behavioral and motivational components of burnout (e.g., reduced efficacy, cynicism) without a commensurate increase in reported exhaustion. Such dissociation has been documented in studies of alexithymia and defensive coping, where emotional awareness is decoupled from physiological or behavioral stress indicators. Thus, the low resilience-low exhaustion group may not genuinely lack exhaustion; rather, their self-reports may underestimate actual depletion due to repressive processes (Campbell-Sills et al., 2006).

From a COR theory perspective, this pattern could be speculatively interpreted as a potential resource transformation deficit. Although the group reports low emotional exhaustion (i.e., resource stock appears intact), their extremely low resilience might impair the ability to mobilize and convert remaining resources into effective coping actions. In COR terms, resource stock alone does not guarantee adaptive outcomes; the capacity to transform resources is a distinct mechanism. If such a deficit exists, even low levels of exhaustion could lead to high burnout because the individual cannot deploy available resources to meet demands. However, we did not directly measure resource transformation capacity, so this interpretation remains hypothetical and requires direct testing in future research. Consistent with this interpretation, training load did not significantly predict membership in this subgroup (*p* = 0.473, see Section 3.6.2), so the pattern is unlikely driven by training volume alone. This subgroup should be interpreted cautiously as a preliminary high-risk type.

The General Type accounted for 89.6% of the population, representing the main group of physical education students. Their resilience and emotional exhaustion levels were close to average, with athletic fatigue and academic burnout at moderate levels, making them a key group for mental health interventions. This proportion is similar to that of the “normative group” in most latent profile studies (Gustafsson et al., 2021; Li et al., 2020; Smith et al., 2018).

The High Resilience-Low Exhaustion type accounted for 5.2%. This group possessed abundant psychological resources, low levels of emotional exhaustion, and significantly lower levels of academic burnout than other groups, reflecting the independent protective role of resilience in academic adaptation. However, there was no significant difference in athletic fatigue between this group and the General Type, possibly because the formation of athletic fatigue is also closely related to objective factors such as training load and physiological recovery (Yang and Ji, 2021), limiting the buffering effect of resilience.

Furthermore, no significant differences in sport type, athletic skill level, or grade were found across profiles. Future research should include measures of coping styles, perceived stress, and objective training load to further validate these profiles and their mechanisms.

### 4.4 Theoretical contributions of integrating variable-centered and person-centered methods

By integrating variable-centered and person-centered research approaches, this study constructed and tested an integrated model of the relationship between athletic fatigue and academic burnout among physical education students, contributing to the following three aspects.

First, it revealed a three-layered structure of “universal mechanism—cross-group consistency—individual differences.” The variable-centered approach (moderated mediation model, multi-group comparison) revealed a universal pattern in the impact of athletic fatigue on academic burnout and its cross-group stability; the person-centered approach (latent profile analysis) identified heterogeneous subgroups of physical education students based on resilience and emotional exhaustion. This integrated research paradigm enables a more comprehensive understanding of complex psychological phenomena (Timans et al., 2023) and systematically addresses the three questions of “what is the mechanism,” “is the mechanism universal,” and “how do individuals differ” within a single study.

Second, it deepened the dual understanding of resilience's function. At the variable-centered level, resilience acted as a moderator, buffering the negative impact of emotional exhaustion on academic burnout; at the person-centered level, resilience served as a classification indicator, jointly forming the core dimensions of psychological risk profiles together with emotional exhaustion. This dual perspective also advances the literature in two ways. First, by applying COR theory to the dual demands of sport and academics, it extends the theory's applicability to cross-domain burnout research among special populations. Second, by integrating a person-centered approach, it identifies the “low resilience-low exhaustion” type as an atypical high-risk group, providing a richer perspective for resilience theory.

Third, building on the integrated approach, this study verified the applicability of the COR theory among physical education students and enriched research on burnout heterogeneity. The identification of the “low resilience-low exhaustion” type as a special risk group challenges the traditional “high exhaustion” risk model, providing a new perspective for burnout research within cultural contexts. The existence of this group suggests that “resource transformation capacity” in COR theory may be a stronger predictor of adaptation outcomes than “resource stock” (Wang et al., 2021), an inference worth further examination in future research.

### 4.5 Limitations and future directions

First, limitations in research design. This study employed a cross-sectional design, which cannot infer causal relationships among variables. Although we have consistently used correlational language throughout, future research could use longitudinal designs (e.g., cross-lagged panel models) to examine dynamic temporal relationships among variables (Smith and Pacewicz, 2022). Specifically, a cross-lagged panel design would help establish the temporal precedence of athletic fatigue to emotional exhaustion and then to academic burnout, which would strengthen causal inferences. Additionally, convenience sampling from a single sport university limits generalizability; future studies should include diverse institutions and regions.

Second, limitations related to measurement and construct overlap. The athletic fatigue (particularly its exhaustion dimension) and emotional exhaustion constructs showed relatively high correlations (*r* = 0.65–0.69). Although we empirically supported discriminant validity via CFA and the Fornell-Larcker criterion, some conceptual overlap remains. Item parceling was used in CFA, which may improve model fit but could mask item-level overlap; a subsequent analysis using all 54 items produced lower fit (CFI = 0.821, RMSEA = 0.063). These constructs may share a general burnout factor, and readers should interpret the structural paths as associative rather than strictly causal. Moreover, our latent profile analysis included only two indicators (resilience and emotional exhaustion), and the low resilience-low exhaustion subgroup was small (5.2%). Replication with larger samples and more indicators is needed before clinical or intervention implications can be drawn. Future research should use item-level analyses and more differentiated measures (e.g., multi-trait multi-method) to further establish distinctiveness.

Third, unmeasured physiological and contextual variables. This study did not collect data on training load, recovery status, sleep quality, overtraining risk, competition phase, or academic workload. These factors may influence athletic fatigue and its spillover to academic burnout and could act as confounders in the hypothesized mediation model. Although we controlled for weekly training hours in the main analyses, more objective and dynamic measures (e.g., session-RPE, heart rate variability, sleep tracking) were not included. Future research should integrate physiological indicators (e.g., session-RPE, heart rate monitoring, sleep tracking) alongside psychological measures such as coping styles and perceived stress, and incorporate these variables as covariates in the moderated mediation model to rule out alternative explanations.

Fourth, common method bias and self-report data. All data were self-reported. Although Harman's single-factor test and the unmeasured latent method factor test indicated that common method bias was within an acceptable range, some bias due to self-reporting (e.g., social desirability, recall) may still exist. Future research could incorporate multi-source data (e.g., coach evaluations, teacher ratings, objective training records). To further control for common method bias, future research may employ a marker variable technique or the CFA marker technique, which can partial out method variance. Collecting multi-source data (e.g., coach ratings of athletic fatigue, official training logs, academic records) would provide convergent validity and reduce same-source inflation.

Fifth, limited variables in the proposed model. This study examined only emotional exhaustion as a mediator and resilience as a moderator. Other important variables (e.g., training engagement, academic self-efficacy, social support, coping styles) were not included and may play significant roles. Future research should develop more comprehensive models.

Future research directions beyond addressing the above limitations include: (1) testing the effectiveness of resilience training or emotion regulation interventions; (2) using multidisciplinary methods (e.g., neuroscience, cognitive psychology) to explore underlying mechanisms; and (3) focusing on special populations (e.g., retired or adolescent physical education students).

## 5 Conclusions

Given the cross-sectional design, all findings are correlational and do not imply causation. The main findings are as follows.

First, athletic fatigue showed a positive association with academic burnout among physical education students. This association was partially mediated by emotional exhaustion. Resilience buffered the link between emotional exhaustion and academic burnout, such that the association was weaker among students with higher resilience. These patterns were consistent across different sport types and athletic skill levels.

Second, physical education students exhibited three distinct profiles based on resilience and emotional exhaustion: a low resilience-low exhaustion type (5.2%), a general type (89.6%), and a high resilience-low exhaustion type (5.2%). The low resilience-low exhaustion group showed the highest levels of athletic fatigue and academic burnout. However, due to the small size of this subgroup (5.2%), these findings should be interpreted with caution. Replication with larger samples and more indicators is needed before clinical or intervention implications can be drawn. No significant differences in sport type, athletic skill level, or grade were found across profiles.

Together, these findings highlight a three-layered structure—universal pattern, cross-group consistency, and individual differences—in the relationship between athletic fatigue and academic burnout. This integrated perspective provides empirical support for targeted interventions while acknowledging the need for replication in larger samples.

## 6 Recommendations

Establish a psychological status monitoring mechanism: Universities should regularly screen physical education students for athletic fatigue, emotional exhaustion, academic burnout, and resilience; establish dynamic psychological records; and focus on identifying the high-risk Low Resilience-Low Exhaustion type group to enable early detection and intervention.

Scientifically regulate training load to alleviate emotional exhaustion: Coaches should reasonably formulate training plans based on the physical and psychological status of physical education students to avoid overtraining-induced emotional exhaustion; strengthen emotional care during training, promptly address negative emotions, and enhance psychological comfort during training.

Systematically cultivate resilience: Integrate resilience training into the regular physical education curriculum, drawing on the “Athlete Resilience Training Guide” (Si et al., 2022). Enhance the resilience levels of physical education students by offering mental health courses, conducting group psychological counseling, teaching cognitive reappraisal and emotion regulation strategies, and setting moderately challenging tasks.

Design standardized and precise stratified intervention programs: Based on the model's generalizability, develop standardized resilience training and emotion regulation programs applicable to all physical education students. Implement precise interventions according to different psychological profiles: For the Low Resilience-Low Exhaustion type, focus on resilience enhancement training while alleviating athletic fatigue and academic burnout; for the General type, enhance resilience through preventive interventions to prevent psychological risks; for the High Resilience-Low Exhaustion type, leverage their role model effect to maintain a good psychological state.

## Data Availability

The original contributions presented in the study are included in the article/Supplementary material, further inquiries can be directed to the corresponding author.

## References

[B1] Aguinis H. Beaty J. C. Boik R. J. Pierce C. A. (2005). Effect size and power in assessing moderating effects of categorical variables using multiple regression: a 30-year review. J. Appl. Psychol. 90, 94–107. doi: 10.1037/0021-9010.90.1.94 15641892

[B2] Campbell-Sills L. Cohan S. L. Stein M. B. (2006). Relationship of resilience to personality, coping, and psychiatric symptoms in young adults. Behav. Res. Ther. 44, 585–599. doi: 10.1016/j.brat.2005.05.001 15998508

[B3] Campbell-Sills L. Stein M. B. (2007). Psychometric analysis and refinement of the Connor-Davidson Resilience Scale (CD-RISC): validation of a 10-item measure of resilience. J. Traumatic Stress 20, 1019–1028. doi: 10.1002/jts.20271 18157881

[B4] Chen F. F. (2007). Sensitivity of goodness-of-fit indexes to lack of measurement invariance. Struct. Equation Modell. 14, 464–504. doi: 10.1080/10705510701301834

[B5] Chen Z. S. Wang H. Y. (2023). Differences and influencing factors of resilience among athletes in different sports. J. Shanghai Univ. Sport 47, 65–73.

[B6] Cohen J. (1988). Statistical Power Analysis for the Behavioural Sciences, 2nd edn. Hillsdale, NJ: Lawrence Erlbaum Associates.

[B7] Connor K. M. Davidson J. R. T. (2003). Development of a new resilience scale: the Connor-Davidson Resilience Scale (CD-RISC). Depression Anxiety 18, 76–82. doi: 10.1002/da.10113 12964174

[B8] Faul F. Erdfelder E. Buchner A. Lang A. G. (2009). Statistical power analyses using G\^*^Power 3.1: tests for correlation and regression analyses. Behav. Res. Methods 41, 1149–1160. doi: 10.3758/BRM.41.4.1149 19897823

[B9] Gustafsson H. Madigan D. J. Lundkvist E. (2021). A latent profile analysis of perfectionism and burnout among Swedish adolescent athletes. Psychol. Sport Exercise 56:102008.

[B10] Halbesleben J. R. B. Neveu J. P. Paustian-Underdahl S. C. Westman M. (2024). Getting to the “COR”: understanding the role of resources in conservation of resources theory. J. Manag. 50, 87–116.

[B11] Hayes A. F. (2013). Introduction to Mediation, Moderation, and Conditional Process Analysis: A Regression-Based Approach. New York, NY: Guilford Press.

[B12] Hobfoll S. E. (1989). Conservation of resources: a new attempt at conceptualising stress. Am. Psychol. 44, 513–524. doi: 10.1037/0003-066X.44.3.513 2648906

[B13] Hu T. Zhang D. Wang J. (2021). A meta-analysis of the trait resilience and mental health: the moderating role of age and culture. Personality Individual Differ. 176:110762.

[B14] Li C. P. Shi K. (2003). The influence of distributive justice and procedural justice on job burnout. Acta Psychol. Sin. 35, 677–684.

[B15] Li N. Wang B. (2023). Cultural characteristics and cultivation pathways of resilience among Chinese physical education students. China Sport Sci. 43, 78–86.

[B16] Li X. Chen Z. S. Zhou Z. X. (2022). Influencing factors and intervention strategies of emotional exhaustion among collegiate athletes. J. Shanghai Univ. Sport 46, 78–87.

[B17] Li Y. Wang J. Zhang Z. X. (2020). The relationship between resilience and academic burnout in physical education students: the mediating role of emotional exhaustion. Chin. J. Sports Med. 39, 789–795.

[B18] Lian R. Yang L. X. Wu L. H. (2005). The relationship between professional commitment and learning burnout of college students and the development of scales. Acta Psychol. Sin. 37, 632–636.

[B19] Liu H. Wang X. Wu D. H. Chen S. (2022). Psychometric properties of the Chinese translated Athlete Burnout Questionnaire: evidence from Chinese collegiate athletes and elite athletes. Front. Psychol. 13:823400. doi: 10.3389/fpsyg.2022.823400 35602744 PMC9120922

[B20] Madigan D. J. Hill A. P. McArthur G. Sorkkila M. (2021). The relationship between athlete burnout and academic outcomes in student-athletes: a systematic review and meta-analysis. J. Sport Exercise Psychol. 43, 501–514.

[B21] Maslach C. Jackson S. E. Leiter M. P. (1996). Maslach Burnout Inventory Manual, 3rd edn. Palo Alto, CA: Consulting Psychologists Press.

[B22] Mazaheri A. H. Abasgholipour A. Tarkhan A. (2025). The effect of sport type and performance level on emotional expressiveness and emotional exhaustion in athletes. J. Motor Control Learn. 7:e162313. doi: 10.5812/jmcl-162313

[B23] Nylund K. L. Asparouhov T. Muthén B. O. (2007). Deciding on the number of classes in latent class analysis and growth mixture modelling: a Monte Carlo simulation study. Struct. Equation Modell. 14, 535–569. doi: 10.1080/10705510701575396

[B24] Podsakoff P. M. MacKenzie S. B. Lee J. Y. Podsakoff N. P. (2003). Common method biases in behavioural research: a critical review of the literature and recommended remedies. J. Appl. Psychol. 88, 879–903. doi: 10.1037/0021-9010.88.5.879 14516251

[B25] Preacher K. J. Kelley K. (2011). Effect size measures for mediation models: quantitative strategies for communicating indirect effects. Psychol. Methods 16, 93–115. doi: 10.1037/a0022658 21500915

[B26] Raedeke T. D. Smith A. L. (2001). Development and preliminary validation of an athlete burnout measure. J. Sport Exercise Psychol. 23, 281–306. doi: 10.1123/jsep.23.4.281 28682196

[B27] Salmela-Aro K. Upadyaya K. (2022). Burnout and engagement across domains: a longitudinal study of school and sport in adolescents. J. Youth Adolescence 51, 891–907.

[B28] Schaufeli W. B. Martínez I. M. Pinto A. M. Salanova M. Bakker A. B. (2002). Burnout and engagement in university students: a cross-national study. J. Cross Cult. Psychol. 33, 464–481. doi: 10.1177/0022022102033005003

[B29] Si G. Y. Zhang Z. Q. Jiang X. B. (2022). Athlete Resilience Training Guide. Beijing: People's Sports Publishing House.

[B30] Smith A. L. Pacewicz C. E. (2022). A longitudinal examination of athlete burnout: testing a cross-lagged panel model. Sport Exercise Perform. Psychol. 11, 45–59.

[B31] Smith A. L. Thomas J. Lee S. (2018). Resilience and burnout in student-athletes: a longitudinal investigation. J. College Stud. Dev. 59, 678–692.

[B32] Timans R. Wouters P. Heilbron J. (2023). Mixed methods research in the social sciences: a review and future directions. Qual. Quan. 57, 2153–2177.

[B33] Vaughan R. S. Laborde S. (2021). Attention, working-memory, and decision-making: the cognitive psychology of open-skill sports. German J. Exercise Sport Res. 51, 312–321.

[B34] Wang J. Li Y. (2022). The relationship between athletic fatigue and academic adjustment among physical education majors: the mediating role of emotion regulation. J. Beijing Sport Univ. 45, 115–124.

[B35] Wang M. Hanges P. J. (2011). Latent class procedures: applications to organisational research. Organ. Res. Methods 14, 24–31. doi: 10.1177/1094428110383988

[B36] Wang Y. Chen X. Gong J. Yan Y. (2021). Resilience and mental health among Chinese university students: a cross-cultural perspective. Int. J. Psychol., 56, 721–729.33340106

[B37] Xi J. Z. Zuo Z. H. (2022). Functional mechanisms and cultivation pathways of resilience: research progress in the past five years. Adv. Psychol. Sci. 30, 1796–1808.

[B38] Yang J. Ji L. (2021). Theoretical models and intervention research progress of athletic fatigue. China Sport Sci. 41, 73–81.

[B39] Ye Y. Wang C. Zhu W. He Y. (2020). Psychometric properties of the 10-item Connor-Davidson Resilience Scale in Chinese college students. J. Affect. Disord. 277, 848–854. 10.1016/j.jad.2019.10.018 31654919

[B40] Zhang H. Liu Y. Wang J. (2024). Latent profile analysis of resilience and emotional exhaustion among physical education students. J. Tianjin Univ. Sport 39, 89–97.

