# Supplementary material for: Athletic fatigue and academic burnout in physical education students: emotional exhaustion, resilience, and latent profiles
Source: Front Psychol. 2026 Jun 19;17:1855670. doi: 10.3389/fpsyg.2026.1855670 (PMC13328198; doi:10.3389/fpsyg.2026.1855670)
Supplement: Supplementary file 1 [file Table_1.docx]

**Multi-group Path Analysis**

1.TITLE: Using Athletic Skill Level as the Grouping Variable

DATA:

FILE = "path_data.csv";

LISTWISE = ON;

VARIABLE:

NAMES = level type abq lb ee res Zee Zres int;

USEVARIABLES = abq lb ee res int;

GROUPING = level (1 = elite 2 = first 3 = second 4 = none);

ANALYSIS:

ESTIMATOR = ML;

BOOTSTRAP = 5000;

MODEL:

ee ON abq (a);

ee ON res;

lb ON ee (b)

abq (cdash)

int (gamma)

res;

abq WITH res;

MODEL CONSTRAINT:

NEW(ind_low ind_med ind_high);

ind_low = a * (b + gamma*(-1));

ind_med = a * b;

ind_high = a * (b + gamma*1);

OUTPUT:

STDYX CINT(bcbootstrap);

MODINDICES(3.84);

2.TITLE: Using Sport Type as the Grouping Variable

DATA:

FILE = "path_data.csv";

LISTWISE = ON;

VARIABLE:

NAMES = level type abq lb ee res Zee Zres int;

USEVARIABLES = abq lb ee res int;

GROUPING = type (1 = open 2 = closed);

ANALYSIS:

ESTIMATOR = ML;

BOOTSTRAP = 5000;

MODEL:

abq (v_abq);

res (v_res);

abq WITH res;

ee ON abq (a);

lb ON ee (b)

abq (cdash)

int (gamma);

lb ON res;

ee ON res;

ee (res_ee);

lb (res_lb);

MODEL CONSTRAINT:

NEW(ind_low ind_med ind_high

total_low total_med total_high

prop_low prop_med prop_high);

ind_low = a * (b + gamma*(-1));

ind_med = a * b;

ind_high = a * (b + gamma*1);

total_low = cdash + ind_low;

total_med = cdash + ind_med;

total_high = cdash + ind_high;

prop_low = ind_low / total_low;

prop_med = ind_med / total_med;

prop_high = ind_high / total_high;

OUTPUT:

STDYX CINT(bcbootstrap);

MOD(3.84);

TECH1 TECH4;

**Latent Profile Analysis**

1.TITLE: LPA 1-class model

DATA:

FILE = "lpa_data.csv";

LISTWISE = ON;

VARIABLE:

NAMES =

id type level grade years

abq lb ee res

Zres Zee;

USEVARIABLES = Zres Zee;

CLASSES = c(1);

ANALYSIS:

TYPE = MIXTURE;

ESTIMATOR = ML;

STARTS = 500 50;

PROCESSORS = 4;

OUTPUT:

TECH11 TECH14;

SAVEDATA:

FILE = "lpa_1class.txt";

SAVE = CPROB;

2.TITLE: LPA 2-class model

DATA:

FILE = "lpa_data.csv";

LISTWISE = ON;

VARIABLE:

NAMES =

id type level grade years

abq lb ee res

Zres Zee;

USEVARIABLES = Zres Zee;

CLASSES = c(2);

ANALYSIS:

TYPE = MIXTURE;

ESTIMATOR = ML;

STARTS = 500 50;

PROCESSORS = 4;

OUTPUT:

TECH11 TECH14;

SAVEDATA:

FILE = "lpa_1class.txt";

SAVE = CPROB;

3.TITLE: LPA 3-class model

DATA:

FILE = "lpa_data.csv";

LISTWISE = ON;

VARIABLE:

NAMES =

id type level grade years

abq lb ee res

Zres Zee;

USEVARIABLES = Zres Zee;

CLASSES = c(3);

ANALYSIS:

TYPE = MIXTURE;

ESTIMATOR = ML;

STARTS = 500 50;

PROCESSORS = 4;

OUTPUT:

TECH11 TECH14;

SAVEDATA:

FILE = "lpa_1class.txt";

SAVE = CPROB;

4.TITLE: LPA 4-class model

DATA:

FILE = "lpa_data.csv";

LISTWISE = ON;

VARIABLE:

NAMES =

id type level grade years

abq lb ee res

Zres Zee;

USEVARIABLES = Zres Zee;

CLASSES = c(4);

ANALYSIS:

TYPE = MIXTURE;

ESTIMATOR = ML;

STARTS = 500 50;

PROCESSORS = 4;

OUTPUT:

TECH11 TECH14;

SAVEDATA:

FILE = "lpa_1class.txt";

SAVE = CPROB;

5.TITLE: LPA 5-class model

DATA:

FILE = "lpa_data.csv";

LISTWISE = ON;

VARIABLE:

NAMES =

id type level grade years

abq lb ee res

Zres Zee;

USEVARIABLES = Zres Zee;

CLASSES = c(5);

ANALYSIS:

TYPE = MIXTURE;

ESTIMATOR = ML;

STARTS = 500 50;

PROCESSORS = 4;

OUTPUT:

TECH11 TECH14;

SAVEDATA:

FILE = "lpa_1class.txt";

SAVE = CPROB;

**Confirmatory Factor Analysis**

1.title:four-factor model CFA.

data:

FILE= "data.csv";

LISTWISE=ON;

VARIABLE:

NAMES = abq_r abq_e abq_d LB_e LB_b LB_a ee001 ee002 ee003 res001 res002 res003;

USEVARIABLES = abq_r abq_e abq_d LB_e LB_b LB_a ee001 ee002 ee003 res001 res002 res003;

ANALYSIS: ESTIMATOR = MLR; ITERATIONS=1000;

MODEL:

abq by abq_r abq_e abq_d;

LB by LB_e LB_b LB_a;

ee by ee001 ee002 ee003;

res by res001 res002 res003;

abq with LB ee res;

LB with ee res;

ee with res;

OUTPUT: SAMPSTAT STDYX MODINDICES(3.84) RESIDUAL;

2.title:three-factor model CFA.

data:

FILE= "data.csv";

LISTWISE=ON;

VARIABLE:

NAMES = abq_r abq_e abq_d LB_e LB_b LB_a ee001 ee002 ee003 res001 res002 res003;

USEVARIABLES = abq_r abq_e abq_d LB_e LB_b LB_a ee001 ee002 ee003 res001 res002 res003;

ANALYSIS: ESTIMATOR = MLR; ITERATIONS=1000;

MODEL:

abqee by abq_r abq_e abq_d ee001 ee002 ee003;

LB by LB_e LB_b LB_a;

res by res001 res002 res003;

abqee with LB res;

LB with res;

OUTPUT: SAMPSTAT STDYX MODINDICES;

3.title:two-factor model CFA.

data:

FILE= "data.csv";

LISTWISE=ON;

VARIABLE:

NAMES = abq_r abq_e abq_d LB_e LB_b LB_a ee001 ee002 ee003 res001 res002 res003;

USEVARIABLES = abq_r abq_e abq_d LB_e LB_b LB_a ee001 ee002 ee003 res001 res002 res003;

ANALYSIS: ESTIMATOR = MLR;

MODEL:

abqLBee by abq_r abq_e abq_d LB_e LB_b LB_a ee001 ee002 ee003;

res by res001 res002 res003;

abqLBee with res;

OUTPUT: SAMPSTAT STDYX MODINDICES;

4.title:single-factor model CFA.

data:

FILE= "data.csv";

LISTWISE=ON;

VARIABLE:

NAMES = abq_r abq_e abq_d LB_e LB_b LB_a ee001 ee002 ee003 res001 res002 res003;

USEVARIABLES = abq_r abq_e abq_d LB_e LB_b LB_a ee001 ee002 ee003 res001 res002 res003;

ANALYSIS: ESTIMATOR = MLR;

MODEL:

abqLBeeres by abq_r abq_e abq_d LB_e LB_b LB_a ee001 ee002 ee003 res001 res002 res003;

OUTPUT: SAMPSTAT STDYX MODINDICES;
