# Supplementary material for: Athletic fatigue and academic burnout in physical education students: emotional exhaustion, resilience, and latent profiles
Source: Front Psychol. 2026 Jun 19;17:1855670. doi: 10.3389/fpsyg.2026.1855670 (PMC13328198; doi:10.3389/fpsyg.2026.1855670)
Supplement: Supplementary file 2 [file Table_2.docx]

**1. Informed Consent Form**

1.1 Research Purpose：This study aims to investigate the training, academic, and psychological status of college physical education majors, and to provide scientific evidence for improving their mental health.

1.2 Research Procedure：You are required to complete a questionnaire, which takes approximately 8–10 minutes. The questionnaire covers aspects such as sports status, academic status, and emotional experiences.

1.3 Risks and Discomforts：This is an anonymous survey without any physical or psychological intervention. It is expected to cause no risks or discomforts.

1.4 Benefits：No direct personal benefits will be provided. However, your participation will contribute to the scientific understanding of the mental health of physical education students.

1.5 Confidentiality：All data will be processed anonymously and used solely for academic research. No personal identity information will be disclosed.

1.6 Voluntary Participation：Your participation is completely voluntary. You have the right to withdraw at any time without any adverse consequences.

1.7 Contact Information：For any questions, please contact the researchers at:

- Phone: 17589518629 - Email: 3196344181@qq.com

1.8 Ethical Statement：This study has been reviewed and approved by the Medical Ethics Committee of Wuhan Sports University.

1.9 Consent Confirmation：By clicking "Start Answering", you indicate that you have read and understood the above content and voluntarily participate in this study. If you do not agree, please close this page directly.

**2. Demographic Information**

1. Gender: ⚪ Male; ⚪ Female

2. Grade: ⚪ Freshman; ⚪ Sophomore; ⚪ Junior; ⚪ Senior

3. Athlete Level: ⚪ National champion; ⚪First-grade; ⚪ Second-grade; ⚪ Unranked

4. Training Years: ⚪ < 3 years; ⚪ 3–6 years; ⚪ 7–10 years; ⚪ > 10 years

5. Weekly Training Hours: ⚪ < 10 hours; ⚪ 10–15 hours; ⚪ 16–20 hours; ⚪ > 20 hours

6. Major Type: ⚪ Physical Education; ⚪ Athletic Training; ⚪ Social Sports; ⚪ Others

7. Sports Skill Type:

⚪Open Skill (e.g., basketball, football, volleyball, table tennis, badminton, tennis, etc.)

⚪Closed Skill (e.g., track and field, swimming, gymnastics, martial arts, shooting, weightlifting, etc.)

**3. Athlete Burnout Questionnaire**

Instructions: Please rate each item based on your actual experience.

(Note: Adjust rating scale as needed; standard 5-point Likert:1=Never, 5=Always)

8. I have done many commendable things in this sport.

9. Training and competition make me so tired that I have no energy to do other things.

10. The effort I spend on training might be better used for other purposes.

11. I feel extremely exhausted during training and competition.

12. I have not achieved much progress in this sport at present.

13. I no longer care about athletic performance as I used to.

14. I feel unable to perform at my usual level.

15. I feel like I am on the verge of collapse.

16. I no longer like this sport as I used to.

17. I feel completely worn out.

18. I no longer care about winning in competitions as I used to.

19. The high physical and mental demands of training and competition exhaust me.

20. No matter how hard I try, I seem unable to reach my potential.

21. I feel that I am successful in this sport.

22. I feel disgusted with this sport.

**4. Academic Burnout Scale for University Students**

Instructions: Please rate each item based on your actual experience.

(Standard 5-point Likert: 1=Completely Disagree, 5=Completely Agree)

23. I have my own learning methods and plans, and I can put them into practice.

24. I think the knowledge I am learning is useless.

25. Mastering professional knowledge is easy for me.

26. When I wake up in the morning, thinking about a day of study makes me feel tired.

27. Instruction: Please select "Completely Disagree" for this item.

28. I find it difficult to maintain long-term enthusiasm for learning.

29. When studying, I can calmly handle emotional issues.

30. After a full day of studying, I feel completely worn out.

31. So far, university study has fully demonstrated my abilities.

32. I am tired of studying.

33. I rarely study after class.

34. I am competent in university courses.

35. I often feel sleepy while studying.

36. I am interested in my major.

37. I feel that my patience in learning is still insufficient.

38. Getting a bachelor's degree is easy for me.

39. I only read books before exams.

40. I want to learn but find it boring.

41. I am full of energy when studying.

42. I rarely plan my study time.

43. Exams always bore me.

**5. Emotional Exhaustion Scale**

Instructions: Please rate each item based on your actual experience.

(Standard 6-point Likert: 1=Never, 6=Every day)

44. Work/study makes me feel physically and mentally exhausted.

45. After a day of work/study, I feel completely worn out.

46. When I wake up in the morning, just thinking about a day of work/study makes me feel terrible.

47. Working/studying all day is really stressful for me.

48. Work/study makes me feel like I am going to collapse.

49. Instruction: Please select "Frequently" for this item.

50. I feel that I have exhausted all my energy.

51. I feel emotionally low while working/studying.

52. I lack enthusiasm while working/studying.

53. I feel that my emotional resources have been depleted.

**6. Resilience Scale**

Instructions: Please rate each item based on your actual experience.

(Standard 5-point Likert: 1=Never, 5=Always)

54. When things change, I can adapt.

55. I can handle anything that happens on the road of life.

56. When facing difficulties, I try to see the positive side.

57. Going through hardships makes me stronger.

58. I can easily recover from illness, injury, or difficulties.

59. I believe I can achieve my goals even when facing obstacles.

60. Under pressure, I can still focus and think clearly.

61. I am not easily defeated by failure.

62. I consider myself a strong person when dealing with life's challenges and difficulties.

63. I can handle unpleasant or painful feelings, such as sadness and anger.
